# Socioeconomic differences in help seeking for colorectal cancer symptoms during COVID-19: a UK-wide qualitative interview study

**DOI:** 10.3399/BJGP.2021.0644

**Published:** 2022-05-31

**Authors:** Athena Ip, Georgia Black, Cecilia Vindrola-Padros, Claire Taylor, Sophie Otter, Madeleine Hewish, Afsana Bhuiya, Julie Callin, Angela Wong, Michael Machesney, Naomi J Fulop, Cath Taylor, Katriina L Whitaker

**Affiliations:** School of Health Sciences, University of Surrey, Guildford.; Department of Applied Health Research, University College London, London.; Department of Targeted Intervention, University College London, London.; London North West University Healthcare NHS Trust, London.; Royal Surrey County Hospital NHS Foundation Trust, Guildford.; Royal Surrey County Hospital NHS Foundation Trust, Guildford.; University College London Hospitals NHS Foundation Trust, London.; Bart’s Health NHS Trust, The Royal Hospital, London.; Bart’s Health NHS Trust, The Royal Hospital, London.; Bart’s Health NHS Trust, The Royal Hospital, London.; Department of Applied Health Research, University College London, London.; School of Health Sciences, University of Surrey, Guildford.; School of Health Sciences, University of Surrey, Guildford.

**Keywords:** colorectal cancer, COVID-19, inequalities, primary care, primary health care, qualitative research

## Abstract

**Background:**

COVID-19 has led to rapid changes in healthcare delivery, raising concern that these changes may exacerbate existing inequalities in patient outcomes.

**Aim:**

To understand how patients’ help-seeking experiences in primary care for colorectal cancer symptoms during COVID-19 were affected by their socioeconomic status (SES).

**Design and setting:**

Qualitative semi-structured interviews with males and females across the UK, recruited using purposive sampling by SES.

**Method:**

Interviews were carried out with 39 participants (20 higher SES; 19 lower SES) who contacted primary care about possible symptoms of colorectal cancer during COVID-19. Data were analysed using framework analysis followed by comparative thematic analysis to explore differences between groups.

**Results:**

Three themes were identified with differences between SES groups: 1) how people decided to seek medical help through appraisal of symptoms; 2) how people navigated services; and 3) impact of COVID-19 on how patients interacted with healthcare professionals. The lower SES group expressed uncertainty appraising symptoms and navigating services (in terms of new processes resulting from COVID-19 and worries about infection). There was also potential for increased disparity in diagnosis and management, with other methods of getting in touch (for example, email or 111) taken up more readily by higher SES patients.

**Conclusion:**

The findings suggest that COVID-19 exacerbated disparities between higher and lower SES participants. This study raises awareness around challenges in help seeking in the context of the pandemic, which are likely to persist (post-COVID-19) as healthcare systems settle on new models of care (for example, digital). Recommendations are provided to reduce inequalities of care.

## INTRODUCTION

Earlier diagnosis of cancer is considered a potential casualty of the COVID-19 pandemic,^1^ leading to avoidable deaths and significant economic impacts.^2^ There have been a number of calls to action to ensure that people seek prompt medical help, and receive appropriate referrals and follow-up care.^3^^,^^4^ This is particularly important in the UK context, given the country’s lower cancer survival compared with countries with similar healthcare systems^5^ and the likely influence of later diagnosis on these differences.^6^ For colorectal cancer, 98% of patients diagnosed at stage I survived their disease for at least 1 year, compared with 44% of patients diagnosed at stage IV.^7^

The impact of COVID-19 on colorectal cancer care is of particular concern because it risks perpetuating persistent social/health inequalities in outcomes,^8^^,^^9^ including late presentation,^10^ less access to treatment,^11^ delayed treatment,^12^ higher mortality,^13^^,^^14^ and poorer survival for more deprived groups.^11^

Evidence from population-based and community studies during the pandemic suggest that people were less likely to seek medical help for their symptoms,^15^^,^^16^ were fearful of catching or transmitting COVID-19 by contacting their GP practice,^16^ and were less likely to be referred, diagnosed, or treated for colorectal cancer.^17^^,^^18^ It is not yet clear whether these impacts are socially patterned.

Recent data from Public Health Scotland suggest that significantly fewer people (25% fewer) were diagnosed with colorectal cancer during the pandemic than before, and there was a trend suggesting that the fall in numbers of people being diagnosed with stage I disease was higher in the most deprived areas compared with less deprived areas.^19^

Primary care underwent rapid transformation as a result of the pandemic,^20^ with 90% of consultations in April 2020 delivered remotely.^21^ These changes present trade-offs for patients^22^ and clinicians,^20^ particularly in the context of assessing potential cancer symptoms.^23^ Any positive consequences (for example, improved flexibility and reducing COVID-19 infection risk) have to be balanced with ensuring adequate diagnostic assessment^23^ and avoiding exacerbation of health inequalities.^24^

This study addressed a gap in the literature by providing qualitative evidence about patients’ experiences of accessing primary care during the pandemic, and whether these experiences were shaped by socioeconomic differences. The study draws on the concept of candidacy as an underpinning framework, as it describes the way in which equity in access can differ because of the way patients and health services determine eligibility for health care.^25^ This evidence can be used to make recommendations to mitigate against exacerbating existing inequalities in care.

**Table:** How this fits in

In order to understand people’s help-seeking behaviours during COVID-19 and inequalities in accessing care, semi-structured interviews were carried out with people who contacted primary care with possible symptoms of colorectal cancer. The Candidacy Framework was used to understand how people decided to seek medical help, how they navigated services, their ease of accessing services, and their perception of the interaction with healthcare professionals. Disparities between higher and lower socioeconomic status participants were found in how people appraised their symptoms during the pandemic, and how they perceived changes in primary care in terms of considerations around the need to visit the GP during the pandemic, understanding how to access primary care, views about remote technology, safety netting during COVID-19, and attitudes towards accessing care. Recommendations are made for minimising negative impacts on patient care during and post-pandemic.

## METHOD

### Approach

Semi-structured interviews were carried out to provide an in-depth understanding of patient experiences of the healthcare system when contacting about possible symptoms of colorectal cancer during the COVID-19 pandemic, and how this varied by socioeconomic status (SES).

### Participant selection and recruitment

Participants were recruited through a research recruitment company (SAROS) who have a database of >60 000 potential responders across the UK and can screen based on different variables to gain populations of interest. Screening questions were developed by the research team to identify people from higher and lower SES groups across the UK who contacted primary care about symptoms related to colorectal cancer during the COVID-19 pandemic (since March 2020). These questions were administered by the recruitment company. After screening, the research company arranged the interview between the participant and the researcher, and provided the researcher with the participant’s contact details. Participants were given £50 for taking part in the interview. Symptoms included in the screening questions were those listed on the Bowel Cancer Awareness Measure toolkit^26^ and Bowel Cancer UK website.^27^ The main index for SES used in this study was education, as this has been used in previous research exploring impact of SES on response to cancer symptoms.^28^ People with O-levels/GCSEs or equivalent or with no formal qualification were in the lower SES group, and those with A-level, or above were in the higher SES group.

### Data collection

Interviews were carried out by an experienced qualitative researcher from October 2020 to November 2020 via phone or Zoom (mean duration = 57 minutes; range = 31–86 minutes). Participants gave verbal consent to take part in the study. Interviews were digitally recorded and transcribed verbatim. The topic guide (Supplementary Box S1) focused on aspects of the Candidacy Framework to understand how people decided to seek medical help, and how they navigated services.^25^

### Analysis

Transcripts were repeatedly read by one qualitative researcher to ensure familiarity with the data, before coding using framework analysis. The Candidacy Framework was used to help organise the data, but themes were developed using an inductive approach. This framework was useful for exploring individuals’ identification of their ‘candidacy’ for accessing and negotiating healthcare services, which highlighted barriers and facilitators to accessing care during the pandemic. Comparative thematic analysis^28^ was then carried out to explore the differences between higher and lower SES groups. This involved initially analysing interviews by socioeconomic group, before moving to analysing differences between groups. This two-step approach to analysis was useful for identifying themes running across the whole sample before looking at the differences between individual groups and comparing them with one another. The lead researcher had multiple data analysis meetings with three members of the team to further refine the findings and ensure that the final themes reflected the data. These were then further discussed with the wider team.

## RESULTS

A total of 39 participants were recruited (mean age = 50 years; range = 25–78 years) from higher (*n* = 20) and lower (*n* = 19) SES backgrounds across the UK. This sample size was to ensure that there were close to equal numbers of participants in both groups so as to be able to conduct a comparative analysis. (For information about participant characteristics including employment status, living arrangements, etc. see Supplementary Table S1.) The most reported symptoms in both groups were a combination of extreme tiredness for no obvious reason and persistent and unexplained changes in bowel habits (*n* = 6). Telephone consultations (*n*, higher SES = 17; *n*, lower SES = 16) were the most reported initial mode of consultation followed by face-to-face (*n*, higher SES = 2; *n*, lower SES = 2), e-consult (*n*, lower SES = 1), and video (*n*, higher SES = 1). Three main themes were identified across the data, exploring:

1. how people decided to seek help;
2. how people navigated services and ease of accessing these; and
3. the impact of COVID-19 on how patients interacted with healthcare professionals (Box 1).

**Box 1.** Summary of differences in responses to possible colorectal cancer symptoms during COVID-19 according to the Candidacy Framework^25^

**Theme (in bold) and subtheme**	**Lower SES**	**Higher SES**
**How people decided to seek medical help through appraisal of symptoms**		
Distinguishing between colorectal and COVID-19 symptoms	Uncertainty in attributing colorectal symptoms to COVID-19	Certainty in differentiating NHS-cited COVID-19 symptoms from colorectal symptoms
Relationship between body vigilance and lifestyle modifications	Less body-vigilant	Heightened body vigilance and ability to connect symptoms to underlying problems

**How people navigated services**		
Accessing health care in the face of a pandemic	Less assertive/confident in accessing primary care compared with others	Described reasons why they were eligible to access primary care
Understanding the process and perceptions of safety measures when accessing primary care	Uncertainty about process and hesitation attending primary care	Realistic expectations of accessing primary care and fewer safety concerns attending primary care

**Impact of COVID-19 on how patients interacted with healthcare professionals**		
Views about utility of remote technology	Reservations about using remote technology	Positive attitudes towards adoption of remote technology
Knowing how and when to seek further help	Reported less active care planning and safety netting	Reported knowing about care planning and safety-netting strategies used

*SES = socioeconomic status.*

### Theme 1. How people decided to seek medical help through appraisal of symptoms

#### Distinguishing between colorectal and COVID-19 symptoms

Some participants in the lower SES group reported uncertainty about whether their colorectal symptoms were associated with COVID-19.^29^ This included their reports of persistent and unexplained changes in bowel habits (for example, diarrhoea), blood in the stool, and extreme tiredness for no obvious reason (which they related to long COVID). For some participants, the idea that their symptoms might be caused by COVID-19 accelerated their decision to consult the GP (see Supplementary Table S2 for details):

*‘I just felt not right and obviously diarrhoea and going to the loo and everything, that was obviously something else that was different, that’s another symptom of the COVID, isn’t it?’*

(Participant [P]5, aged 51 years, lower SES, extreme tiredness for no obvious reason, unexplained changes in bowel habits)

Their reasons for contacting the GP were mainly to assure themselves of the right course of action, for example, whether they should go for a COVID-19 test. Some expressed uncertainty about whether their symptoms could be attributed to COVID-19:

*‘I first thought have I got coronavirus, I thought but you just you know, your mind just goes crazy with it and I thought I’ve never heard anything on the news that people get pains in their stomach if they’ve got coronavirus, but you think you’ve got it because you’re poorly, you know what I mean?’*

(P10, aged 46 years, lower SES, pain in abdomen)

In contrast, people in the higher SES group were more confident in the likelihood that their symptoms were related to COVID-19, which influenced their assessment of going to the GP practice:

*‘Obviously with a stomach thing it’s less likely that I’ve got COVID, so I suppose they’re more amenable to you coming in.’*

(P21, aged 61 years, higher SES, pain in abdomen, persistent and unexplained changes in bowel habits)

#### Relationship between body vigilance and lifestyle modifications

Some people in the higher SES group reported how lifestyle modifications during the pandemic, including working from home or being furloughed, made them more vigilant about changes in their body. They reported how this increased awareness was a driver for seeking help from the GP:

*‘I didn’t wait as long as what I would have done in the past, probably because then I had to start working from home and teach from home and all that so I felt like I had more control of my time.’*

(P17, aged 54 years, higher SES, rectal bleeding or blood in stool, persistent and unexplained changes in bowel habits)

Changes in diet and exercise, as well as heightened worry and stress, were described by he higher SES group as contributing to their symptoms and may have made symptom discrimination more difficult:

*‘I thought, maybe the weight loss was because we are at home, we all went for exercise, healthier eating, cooking more at home, you know.’*

(P16, aged 50 years, higher SES, unexplained weight loss, pain in abdomen)

*‘Well I wondered at first whether it might be, you get a bit anxious in lockdown and so on and I’m a vulnerable age, so I thought it might be that, but then I wasn’t so sure.’*

(P28, aged 70 years, higher SES, persistent and unexplained changes in bowel habits)

In contrast, people in the lower SES group did not mention lifestyle changes, working from home, or being furloughed as influences on whether they noticed symptoms or sought help from their GP.

### Theme 2. How people navigated services

#### Accessing health care in the face of a pandemic

Both groups weighed up the balance between their own needs against potential risks and NHS resources. However, people in the higher SES group appeared to be more determined to contact their GP for advice, despite the pandemic, in case their condition was serious or worsened:

*‘Lockdown didn’t influence my decision at all. I made the decision because I needed some clinical advice and action.’*

(P4, aged 67 years, higher SES, rectal bleeding or blood in stool)

*‘Because I kind of like understand that COVID and stuff like that, there is more danger, obviously, than usual. But in the same time, I was concerned about my health, so for me it doesn’t matter, I was determined to see GP or, you know, kind of like book an appointment, to be honest. So it was in back of my mind, the concern, but it will, it will not stop me kind of like to book an appointment, to be honest.’*

(P16, aged 50 years, higher SES, unexplained weight loss, pain in abdomen)

People in the lower SES group showed more hesitancy in accessing health care during the pandemic. They showed particular concern about what to expect, comparing themselves with others who may have been worse off, and not wanting to waste NHS resources:

*I’d have probably left it for another few months and seen how it went on sort of thing. I know, I know the pandemic’s on and there’s people suffering a lot worse than me and what have you but the GPs should still be there sort of on the ground floor to sort out basic ailments and illnesses and what have you.’*

(P8, aged 60 years, lower SES, extreme tiredness for no obvious reason, persistent and unexplained changes in bowel habits)

As a result, there was evidence that people with lower SES may have delayed seeking help longer than participants in the higher SES group:

*‘I didn’t want to really go into a doctor’s, go into a hospital, if I didn’t have to do, which is why I you know, initially sort of tried to put it off and you know, ignored it a bit when it first started …’*

(P39, aged 33 years, lower SES, extreme tiredness for no obvious reason, pain in abdomen, persistent and unexplained changes in bowel habits)

#### Understanding the process and perceptions of safety measures when accessing primary care

Both groups expected that the process of getting an appointment would be different because of COVID-19. However, people in the higher SES group appeared to know more about what to expect when deciding to access primary care during the pandemic. This included expectations of contacting the practice by phone and expecting telephone consultations initially, with a better understanding of the triage process in general:

*‘I knew that they were doing these telephone consultations, but I felt that my condition was nothing you could do over the telephone or video, and that it would have to be a visit …’*

(P4, aged 67 years, higher SES, rectal bleeding or blood in stool)

Some people in the lower SES group had mixed views about what to expect from accessing services during the pandemic, thinking that it would be the same as pre-pandemic, while others assumed that services would not be available except in case of emergency and that telephone lines would be busier:

*‘I thought I was actually going to see the doctor the first time but I thought they was just going to say to you wear a mask, infection, you know.’*

(P6, aged 36 years, lower SES, extreme tiredness for no obvious reason, rectal bleeding or blood in stool, persistent and unexplained changes in bowel habits)

*‘I didn’t think they’d be overrun with patients or full sort of working, I just thought they might be shut and only emergencies go through the doctors.’*

(P8, aged 60 years, lower SES, extreme tiredness for no obvious reason, persistent and unexplained changes in bowel habits)

However, people in both groups expressed hesitancy about attending their GP practice because of fears of catching COVID-19:

*‘But unless it’s necessary, I’d still rather avoid it.’*

(P12, aged 32 years, lower SES, pain in abdomen, rectal bleeding or blood in stool)

Higher SES participants also reported trust in the system/themselves to avoid infection, which meant they were more confident to attend:

*‘The measures that all doctors’ practices will be taking, and similar services, will be very good and will be efficient and effective. So I’m quite confident in what they would be able to do to protect you from COVID.’*

(P4, aged 67 years, higher SES, rectal bleeding or blood in stool)

This indicates that having confident expectations about making contact with health services and avoiding contagion could affect help-seeking behaviours, particularly disadvantaging lower SES participants.

### Theme 3. Impact of COVID-19 on how patients interacted with healthcare professionals

#### Views about utility of remote technology

The majority of people in the higher SES group and some in the lower SES group described advantages to remote consultations such as convenience (higher and lower SES), not competing with work commitments (lower SES), and recognising that the use of technology was a positive outcome of the pandemic and the way forward for the NHS (higher SES):

*‘So I think maybe it’s speeded up the technology for the better that will enable GPs to maximise their time.’*

(P33, aged 75 years, higher SES, pain in abdomen, rectal bleeding or blood in stool, persistent and unexplained changes in bowel habits)

*‘*[…] *it’s a lot quicker and it is a lot more convenient, you know, like I say, being able to maybe contact the GP and get an appointment from work, rather than having to take you know a couple of hours out of work, or an hour out of work to actually go down …’*

(P39, aged 33 years, lower SES, extreme tiredness for no obvious reason, pain in abdomen, persistent and unexplained changes in bowel habits)

A few people in the higher SES group had reservations about remote consultations because they felt that it could not be used to resolve symptoms, it was difficult to read emotions and facial expressions, and required existing ‘ *face-to-face established relations’* (P27, aged 50 years, higher SES, extreme tiredness for no obvious reason, persistent and unexplained changes in bowel habits).

However, more than half of the participants in the lower SES group described reservations, including practical barriers such as missing phone calls and not getting a call back, dependence on internet/technology working, and additional disadvantages for specific groups such as older people:

*‘But if you don’t answer the phone in two or three rings, you’re cancelled. That’s the disadvantage of it* […] *Not the way you’d want to do it every time once the pandemic’s over.’*

(P5, aged 51 years, lower SES, extreme tiredness for no obvious reason)

*‘People that live near me they’re all in their 80s and 90s and what have you, they don’t even know how a mobile phone works, they keep one at the side of them if they need one for emergencies.’*

(P8, aged 60 years, lower SES, extreme tiredness for no obvious reason, persistent and unexplained changes in bowel habits)

Some people reported that they would prefer video consultations if offered as the health professional would be able to see the symptom as opposed to over the telephone where it can be more difficult to explain:

*‘I prefer face-to-face, even if you’re not in direct contact, at least they can see you. Because trying to sometimes explain over the phone your symptoms is sometimes a bit difficult.’*

(P6, aged 36 years, lower SES, extreme tiredness for no obvious reason, rectal bleeding or blood in stool, persistent and unexplained changes in bowel habits)

#### Knowing how and when to seek further help

The increase in remote contact methods for primary care affected how lower and higher SES groups felt their safety was being monitored. For example, people in the higher SES group reported more active forms of safety netting, including a timescale for re-contact when symptoms had not resolved, medication to ease symptoms while waiting for their referral appointment, or other remote options of getting back in touch such as email or calling 111. This made participants feel reassured:

*‘There was the reassurance that you know, if this, if the symptoms persist then you will need to call me back, because we might need to investigate it a bit further.’*

(P11, aged 52 years, higher SES, persistent and unexplained changes in bowel habits)

People in the lower SES group had mixed perceptions about how they were monitored, with some participants mentioning not being given safety-netting advice and others not remembering what advice was given:

*‘After the “it’s probably not COVID” I’m not going to lie to you I kind of mentally checked out a bit, I was like oh cool, I don’t have to worry then …’*

(P12, aged 32 years, lower SES, pain in abdomen, rectal bleeding or blood in stool)

Another participant felt that a follow-up appointment was less likely because of COVID-19:

*‘I mean, it does feel different to what it used to, where you’d always get a follow-up, always, this time I don’t feel that you will, necessarily.’*

(P23, aged 56 years, lower SES, pain in abdomen)

## DISCUSSION

### Summary

Interviews with the public who contacted primary care during the pandemic highlighted disparities between SES groups in how people decided to seek medical help, how they navigated services, and how they perceived interactions with healthcare professionals. The overshadowing of COVID-19 and uncertainty about its symptoms had different effects on both groups. Compared with the lower SES group, the higher SES group appeared more certain about signs of COVID-19 and were more likely to seek a face-to-face appointment and follow-up consultations for their colorectal symptoms, rather than worrying about viral transmission. Furthermore, lifestyle changes because of COVID-19 restrictions allowed people in the higher SES group to be more body-vigilant, which appeared to speed up their decision to consult. Awareness of the NHS being under strain and the increased needs of the public may have had a differential effect on the groups in terms of prioritising their own health needs, as the lower SES group appeared more concerned with burdening the NHS. Perceptions around accessibility and safety measures during the pandemic were divided according to SES, with the higher SES group knowing more about how to navigate access than the lower SES group. Differences were also found in attitudes towards remote consultations, with more reservations reported by people in the lower SES group, which may have had an impact on participants’ ability to articulate their symptoms and may have implications for future help-seeking behaviours. Finally, with the introduction of remote consultations during the pandemic there was an increased importance of transparent safety netting and care planning, and differences in the use/perceptions of these may have exacerbated inequalities between higher and lower SES participants.

### Strengths and limitations

To the authors’ knowledge, this is the first study to explore views and experiences of patients accessing primary care during the pandemic for symptoms of colorectal cancer, and whether these views varied between socioeconomic groups. Participants were recruited from across the UK, making the findings generalisable to other UK nations.

Participants were identified through a research company, a method of recruitment that can result in a highly self-selecting group of participants who have a degree of technical skill that may increase their competence in engaging with telemedicine during COVID-19 restrictions. However, it also increases access to people from lower SES groups who may otherwise be hard to reach and recruit.

The means by which participants were recruited meant that member checking following study completion was not possible, although previous research in the field has found this to be a helpful strategy for augmenting and interpreting interview data with participants.^30^

Although participants were sampled into higher and lower SES groups to draw out differences, the binary nature of the analyses is a limitation, and does not necessarily capture the nuances across the SES gradient. The analysis also focused on people who sought help during the pandemic, as there was a particular interest in understanding how changes owing to the pandemic influenced access to primary care. However, it would have been useful to also capture views of those who did not seek help. Finally, this research did not capture healthcare professionals’ views on how delivery of care changed and the challenges communicating this to patients; however, this will be explored in another study.

The mean age of the sample was reflective of when the risk of colorectal cancer starts to rise in the general population,^31^ although those in the higher SES group were on average slightly older than those in the lower SES group, which may have impacted aspects of the pathway (for example, decision to refer). However, it remains important to ensure safety-netting strategies and communication about next steps are the same for all patient subgroups, particularly as the incidence of colorectal cancer is rising in younger age groups.^32^

### Comparison with existing literature

These findings support previous research, which applied the Candidacy Framework to understand how aspects of the doctor–patient interaction influence perceived eligibility for help seeking. This is based on challenges with recognising that symptoms need medical attention, and subsequently, how to navigate services.^25^ However, this work extends the findings by showing differences between lower and higher SES groups. This study found that the higher SES group reported being more body-vigilant during the pandemic and were more likely to seek help, linking this to working from home or being furloughed. This is in line with a previous study exploring the impact of body vigilance on cancer ‘alarm’ symptoms,^33^ which found that paying more attention to bodily changes was significantly associated with seeking help for cancer symptoms. This was less apparent in the lower SES group.

People in the lower SES group were less certain when discussing the cause of their symptoms and were more likely to worry that their symptoms were COVID-19 related (which would make them more likely to consult their GP about COVID-19 and risk delaying care for potential symptoms of colorectal cancer). Previous studies have also found that non-recognition of symptom seriousness and less knowledge of cancer symptoms in lower educated groups^34^^–^^37^ is linked to delays in presentation for lower SES groups.^38^^,^^39^

The finding that people in the lower SES group were more likely to express concern about burdening the healthcare system may be explained within the context of candidacy, as people in more deprived areas may witness a greater burden of ill health in their communities, and therefore higher levels of frequent attendance in primary care.^40^

Safety netting during the pandemic is also highlighted as important in a previous study, as it can be an effective way of monitoring people to reduce clinical risk in remote consultations and in situations of uncertain clinical presentations.^41^ However, patients need to know how to seek further help/re-present if their symptoms persist. This is of particular significance since it is known that COVID-19 has led to delayed presentations, suggesting that people require additional support when navigating through the pathway owing to new methods of contacting the GP.^3^

Many of the participants in this study experienced remote consultations by telephone, as opposed to video, despite some participants reporting that they would have liked a video consultation if given the choice. However, the use of video consultations is rare in general practice as there is a perception that telephone or face-to-face consultations are more useful for the majority of circumstances.^21^ The importance of considering patient preferences when offering remote consultations and recognising how it may be difficult for certain groups was echoed in a recent study exploring experiences of healthcare professionals during the pandemic.^20^ Other studies have also mentioned how remote consultations could potentially widen inequalities in health care, particularly for older and vulnerable groups.^24^^,^^42^^–^^44^

This study found that participants navigated services during the pandemic depending on their perceptions of safety and knowledge of processes (for example, online appointment systems). This is consistent with a recent survey study that found there was low public awareness of changes to face-to-face consultations.^45^ Reluctance to access primary care owing to fears of catching COVID-19 is reported by other studies conducted around the pandemic.^3^^,^^46^^,^^47^ Addressing barriers to accessing care may help to reduce this gap,^48^ which was already a concern pre-pandemic.

### Implications for research and practice

A summary of recommendations for practice is provided in Box 2. With the uncertainty around COVID-19 symptoms and colorectal cancer symptoms, there is the potential for future impact of long COVID on colorectal symptom appraisal and confusion.^49^ Furthermore, the changing nature of guidance about COVID-19 symptoms and differences between NHS and other public bodies in their public-facing communication (for example, where bowel symptoms are present in some but not others) makes raising awareness of symptoms a challenging avenue. There needs to be consensus on advice that is accessible to the public to avoid ongoing confusion and disseminating these through campaigns and more sustainable information (for example, on GP practice websites and NHS letters).

**Box 2.** Recommendations for practice

Provide accurate and up-to-date information about symptoms of COVID-19 in healthcare settings and on relevant websites. Build on campaigns designed to promote symptom awareness and importance of earlier cancer diagnosis, to specifically target sociodemographic groups less likely to recognise symptoms of colorectal cancer or less likely to be vigilant about changes in their bodies. Provide better signposting to services and pathways to access these by utilising known, effective ways to communicate new bookings and consulting methods with patients, as well as keeping GP surgery websites and their phone/text communication up to date. Ensure infection control measures for COVID (and wider) are overt and embedded into NHS services. Provide people with active safety netting, including a timeframe for follow-up or symptoms to look out for and ensure that follow-up options (for example, patient-activated call, GP app access) are offered to all patients, who are provided with help to use it or alternative options if needed. Support and build on training for primary care to address health inequalities, particularly around access and digital exclusion.

There is an urgent need for campaigns to encourage lower SES groups in particular to notice changes in their bodies, such as offering practical action plans (for example, campaigns to encourage people to set aside time to notice any changes and provide flexible and feasible options to make appointments), as external influences (such as type/place of employment) may make it harder for them to notice and interpret potential cancer symptoms. For people in the higher SES group this will also be important as the easing of the pandemic will result in more people going back to work and being less likely to notice bodily changes.

Reassuring people that safety measures are in place through reliable sources, and using different methods to reach people (email, text, letter, practice website) will help, particularly for people from lower SES groups who showed more hesitancy around accessing primary care owing to uncertainty about the process and subsequent safety measures.

It is important that people are signposted to appropriate and reliable information about methods of consultation. Proactive efforts to address primary care access and ensuring that patients have an option for face-to-face or remote consultation will support those with digital access challenges.

Providing people with active safety netting through different methods (for example, providing people with a timeframe of when to reconsult and through different methods such as phone, email, or app) will help going forward, especially given the backlog in cancer referrals^17^ and the added uncertainty owing to the pandemic.

Further research is needed to look at how inequalities may be generated across the care pathway, gathering perceptions from healthcare professionals on the main changes to the cancer pathway and their impact on inequalities, and also to link changes in care with data (similar to epidemiological studies conducted during COVID-19)^1^^,^^8^ to see whether the gap is sustained. For example, concerns have been raised that the introduction of quantitative faecal immunochemical test (qFIT) to stratify risk and prioritise patients for limited endoscopic services has led to missed cancers,^50^ despite showing promise pre-pandemic.^51^

In conclusion, this study provides important insights into barriers accessing primary care during the pandemic and highlights the disparities between higher and lower SES groups including appraisal of symptoms, perceptions around safety and availability of services during the pandemic, use of remote consultations, and safety netting. Recommendations are provided on how these inequalities can be reduced and barriers that may lead to delayed cancer diagnosis potentially decreased. These findings may also be applicable to help seeking for other symptoms, not just those related to colorectal cancer.
